# A Semi‐Crystalline Polymer Semiconductor with Thin Film Stretchability Exceeding 200%

**DOI:** 10.1002/advs.202302683

**Published:** 2023-05-25

**Authors:** Yejin Kim, Hyungju Ahn, Dahyeon Yoo, Mingi Sung, Hyeonjin Yoo, Sohee Park, Junghoon Lee, Byoung Hoon Lee

**Affiliations:** ^1^ Department of Chemical Engineering and Materials Science Graduate Program in System Health Science and Engineering Ewha Womans University Seoul 03760 Republic of Korea; ^2^ Pohang Accelerator Laboratory POSTECH Pohang 37673 Republic of Korea; ^3^ Division of Chemical Engineering Dongseo University Busan 47011 Republic of Korea

**Keywords:** bimodal crystalline structure, fluorination, intrinsically stretchable polymer semiconductors, stretchable electronics, thin film stretchability

## Abstract

Despite the emerging scientific interest in polymer‐based stretchable electronics, the trade‐off between the crystallinity and stretchability of intrinsically stretchable polymer semiconductors—charge‐carrier mobility increases as crystallinity increases while stretchability decreases—hinders the development of high‐performance stretchable electronics. Herein, a highly stretchable polymer semiconductor is reported that shows concurrently improved thin film crystallinity and stretchability upon thermal annealing. The polymer thin films annealed at temperatures higher than their crystallization temperatures exhibit substantially improved thin film stretchability (> 200%) and hole mobility (≥ 0.2 cm^2^ V^−1^ s^−1^). The simultaneous enhancement of the crystallinity and stretchability is attributed to the thermally‐assisted structural phase transition that allows the formation of edge‐on crystallites and reinforces interchain noncovalent interactions. These results provide new insights into how the current crystallinity–stretchability limitation can be overcome. Furthermore, the results will facilitate the design of high‐mobility stretchable polymer semiconductors for high‐performance stretchable electronics.

## Graphical abstract

An intrinsically stretchable polymer semiconductor with thin film stretchability exceeding 200% is developed by tailoring its crystalline structure. The polymer semiconductor exhibits simultaneously enhanced crystallinity, charge‐carrier mobility, and stretchability, for the first time, through strengthened noncovalent interactions in dynamically oriented edge‐on crystallites under tensile strain, strongly holding adjacent amorphous domains.

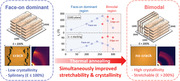

## 1 Introduction

Intrinsically stretchable polymer semiconductors (ISPSs), which are electrically conductive and malleable under mechanical strain without using additives,^[^ ^1^, ^2^, ^3^, ^4^ ^]^ elastomers,^[^ ^5^, ^6^, ^7^, ^8^ ^]^ and flexible linkers,^[^ ^9^, ^10^ ^]^ are key elements in polymer‐based stretchable electronics.^[^ ^11^, ^12^, ^13^, ^14^, ^15^, ^16^, ^17^ ^]^ To develop high‐performance stretchable electronics, the ISPSs should have high charge‐carrier mobility (*µ*) and high stretchability.^[^ ^11^, ^12^, ^13^, ^18^, ^19^, ^20^ ^]^ However, the ISPSs undergo a trade‐off between crystallinity and stretchability; generally, polymer semiconductors with enhanced crystallinity can increase the *µ* but are splintery upon mechanical stretching.^[^ ^21^, ^22^, ^23^, ^24^, ^25^ ^]^ This trade‐off substantially restricts the design of diverse materials for high‐performance and reliable ISPSs; therefore, the design strategy has been limited to the development of low‐crystalline polymer semiconductors, which may exhibit moderate *µ*.^[^ ^23^, ^24^, ^25^ ^]^ Although a few ISPSs with reduced crystallinity showed high *µ* and stretchability,^[^ ^13^, ^15^, ^26^ ^]^ a new design strategy for ISPSs with high crystallinity and stretchability can allow the reconsideration of the current crystallinity–stretchability trade‐off. Moreover, the new design strategy should allow the development of more diverse high‐mobility and stretchable ISPSs using numerous donor and acceptor moieties already developed for high‐mobility semi‐crystalline polymer semiconductors.

The thin film stretchability of the semi‐crystalline polymer semiconductors is lower than that of their amorphous counterparts. Researchers have demonstrated the crystallinity–stretchability trade‐offs; the semi‐crystalline polymer semiconductors composed of 3‐hexylthiophene and cyclopentadithiophene‐*co*‐benzothiadiazole, with increasing regioregularity and crystallinity, exhibit poor stretchability.^[^ ^22^, ^23^, ^26^ ^]^ Therefore, they have not been considered for stretchable electronics despite the numerous materials developed. Additionally, as the crystallinity–stretchability relationship is not yet fully understood, developing high‐mobility ISPSs with high crystallinity and high stretchability remains challenging.^[^ ^19^, ^20^ ^]^

Herein, we report a highly stretchable polymer semiconductor based on cyclopentadithiophene (CDT) and fluorinated benzotriazole (BTA) moieties, and it shows simultaneously improved crystallinity and stretchability enabled by tailoring its thin film crystalline structure. Compared with the reference polymer without the fluorination of the BTA unit (poly(4‐(5‐(4,4‐dihexadecyl‐4H‐cyclopenta[1,2‐b:5,4‐b″]dithiophen‐2‐yl)thiophen‐2‐yl)‐2‐octyl‐7‐(thiophen‐2‐yl)‐2H‐benzo[d][1,2,3]triazole) (PCDTBTA); P1), the fluorinated counterpart (poly(4‐(5‐(4,4‐dihexadecyl‐4H‐cyclopenta[1,2‐b:5,4‐b″]dithiophen‐2‐yl)thiophen‐2‐yl)‐5,6‐difluoro‐2‐octyl‐7‐(thiophen‐2‐yl)‐2H‐benzo[d][1,2,3]triazole) (PCDTFBTA); P2) undergoes a structural phase transition from face‐on dominant to bimodal (mixed face‐on/edge‐on) crystalline phase upon thermal annealing. Annealed P2 thin films with a thickness of 50 nm showed simultaneously enhanced crystallinity, *µ*, and stretchability, with the unprecedented crack onset strain (*ε*_c_) exceeding 200%. To the best of our knowledge, our PCDTFBTA polymer shows the highest thin film stretchability for ISPSs, with concurrently improved crystallinity and stretchability, thereby overcoming the current crystallinity–stretchability limitation.

## 2 Results and Discussion

### 2.1 Thermally‐Assisted Structural Phase Transition

**Figure 1:**
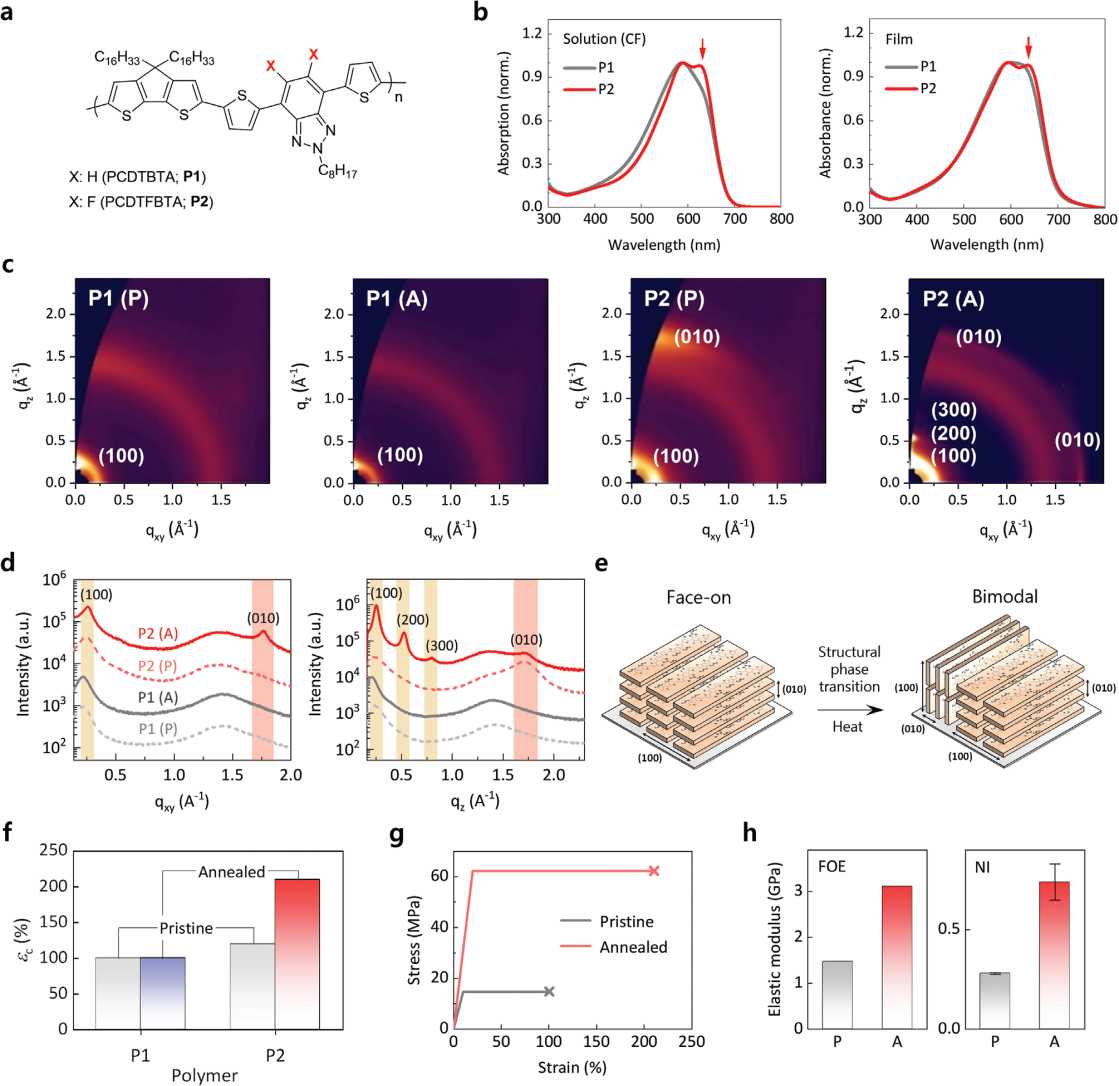
Thermally‐assisted structural phase transition. a) Chemical structures of P1 and P2. b) Normalized UV–vis absorption spectra of the P1 and P2 solutions and thin films. The red arrows indicate absorption peaks corresponding to 0–0 electronic transitions. c) GIWAXD reciprocal space maps for the P1 and P2 thin films before (P) and after (A) thermal annealing, where the *q*_xy_ and *q*_z_ indicate the IP and OOP directions, respectively. d) Corresponding linecut profiles in the IP (left) and OOP (right) directions extracted from the reciprocal space maps shown in c. e) Schematic representation of the structural phase transition of the P2 thin films enabled by thermal annealing. f) *ε*_c_ values of the P1 and P2 thin films before (pristine) and after (annealed) thermal annealing. g) Stress–strain curves of the P2 thin films before (pristine) and after (annealed) thermal annealing at 270 °C, where the yield point, elastic modulus, and fracture point (i.e., *ε*_c_) were obtained from the film‐on‐elastomer (FOE) results (Figures S4, S5, and S7, Supporting Information). h) Elastic moduli of the P2 thin films before and after thermal annealing at 270 °C were obtained following the FOE and NI experiments, respectively.

We prepared the P1 and P2 polymers by a Stille coupling reaction (**Figure** **1**a; Scheme S1, Supporting Information).^[^ ^27^ ^]^ Figure 1b shows the normalized ultraviolet–visible (UV–vis) absorption spectra of P1 and P2 solutions and thin films. P1 and P2 exhibited strong intramolecular charge transfer bands in the spectral range of 450–700 nm. However, P2 showed additional 0–0 mode peaks at *λ*_max_ of 625 and 635 nm for the solutions and thin films, respectively, implying strong *π*–*π* interactions between the polymer chains.^[^ ^28^, ^29^ ^]^ Therefore, higher crystallinity and *µ* were expected for P2 than for P1 because of the F···S noncovalent interaction‐induced coplanar backbone.^[^ ^30^ ^]^

The relatively high crystallinity of P2 was verified by the 2D grazing incidence wide‐angle X‐ray diffraction (GIWAXD) measurements of the P1 and P2 thin films. As shown in the reciprocal space maps, linecut profiles, and crystallographic parameters for the P1 and P2 thin films before and after the thermal annealing (Figure 1c,d; Table S1, Supporting Information), the pristine P1 thin film exhibited an amorphous feature with a halo in *q* = 1.0–2.0 Å^−1^. This featureless diffraction pattern was maintained even after the thermal annealing at 150 °C. In contrast, the pristine P2 thin film exhibited *π*–*π* stacked polymer crystallites preferentially in the face‐on configuration. The crystallinity was improved after the thermal annealing at 270 °C, confirmed by the significantly increased crystalline length (*L*_c_) in the in‐plane (IP; *q*_xy_) and out‐of‐plane (OOP; *q*_z_) directions. Note that these optimal annealing temperatures were determined by finding the maximum *µ*. Further, the annealed P2 thin film showed a lower relative degree of crystallinity (rDoC) than the previously reported crystalline CDT‐based polymer, poly[(5‐fluoro‐2,1,3‐benzothiadiazole‐4,7‐diyl)(4,4‐dihexadecyl‐4H‐cyclopenta[2,1‐b:3,4‐b″]dithiophene‐2,6‐diyl)(6‐fluoro‐2,1,3‐benzothiadiazole‐4,7‐diyl)(4,4‐dihexadecyl‐4H‐cyclopenta[2,1‐b:3,4‐b″]dithiophene‐2,6‐diyl)] (PCDTFBT), which is not stretchable (Figure S1, Supporting Information).^[^ ^22^ ^]^ More importantly, we observed a structural phase transition for the P2 thin film from a face‐on‐dominant to a bimodal configuration after the thermal annealing, as illustrated in Figure 1e, regardless of the processing solvents (Figure S2 and Table S2, Supporting Information).

### 2.2 Simultaneous Enhancement of the Crystallinity and Stretchability

To investigate the crystallinity–stretchability relationship of P1 and P2, we performed *ε*_c_ measurements on the pristine and annealed P1 and P2 thin films before and after tensile stretching at various strains (Figure S3, Supporting Information). The pristine P1 thin films showed *ε*_c_ values of 100%. This value was unchanged after the thermal annealing (Figure 1f; Figure S4, Supporting Information). The pristine P2 thin films yielded similar *ε*_c_ values (≈120%); however, the annealed P2 thin films showed remarkably improved *ε*_c_ exceeding 210% (Figure S5, Supporting Information). Considering the enhanced crystallinity of the P2 thin films after the thermal annealing (Figure 1c,d), the simultaneously enhanced crystallinity and stretchability of the annealed P2 thin films were surprising results. Since the P2 thin films showed a negligible change in the film thickness after the thermal annealing (Figure S6, Supporting Information), the enhancement of the *ε*_c_ of the annealed P2 thin films can be explained by the variation in their crystalline structures (Figure 1e).

The mechanical properties of the pristine and annealed P2 thin films were investigated by film‐on‐elastomer (FOE) and nanoindentation (NI) experiments. The optical microscopy (OM) images of the pristine and annealed P2 thin films, which were stretched and subsequently released to the original position, revealed their critical buckling strain (*ε*_cr_), where *ε*_cr_ is the lowest tensile strain that generates buckles on the polymer thin films (Figure S7, Supporting Information). The elastic moduli, *ε*_c_, and *ε*_cr_ of the pristine and annealed P2 thin films determined from the FOE results were used to complete their stress–strain curves (Figure 1g). The annealed P2 thin film showed enhanced stretchability with an increased yield point, tensile strength, and fracture point. The enhanced elastic modulus of the P2 thin films from 1.47 to 3.11 GPa after the thermal annealing was verified by the NI results, yielding a similarly enhanced (approximately two‐fold increase) average elastic modulus from 0.28 to 0.74 GPa after the thermal annealing (Figure 1h).

### 2.3 Temperature‐Dependent Stretchability

**Figure 2:**
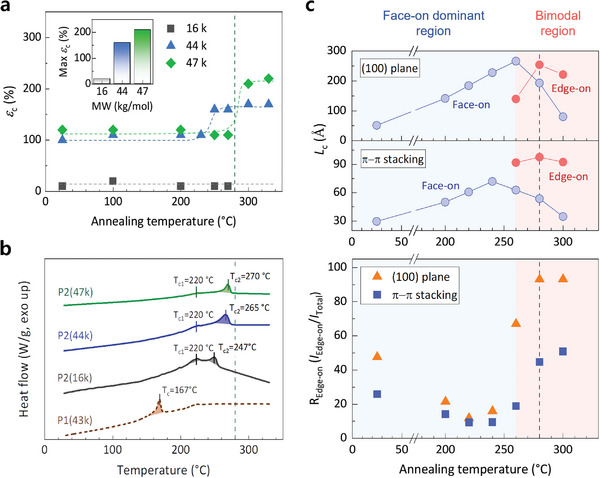
Temperature and MW‐dependent stretchability. a) the *ε*_c_ variations of the P2 thin films with various MWs (16, 44, and 47 kg mol^−1^) as a function of the annealing temperature, where the inset shows the maximum *ε*_c_ values of various P2 polymers. b) The DSC curves of P1 and P2 with various MWs. c) The *L*_c_ variations of the face‐on and edge‐on crystallites (top) and edge‐on crystallite ratio (bottom) of P2 with a MW of 47 kg mol^−1^ as a function of the annealing temperature. The green lines at 280 °C indicate the *T*_s_ for P2 with a MW of 47 kg mol^−1^.

The stretchability enhancement of P2 strongly depended on its molecular weight (MW) and annealing temperature. We measured the *ε*_c_ of the P2 thin films with various number‐average MWs (16–47 kg mol^−1^) in the full range of annealing temperatures (25°C–340°C) (**Figure** **2**a). Note that P2 with a MW of 47 kg mol^−1^ was used throughout the study except for this experiment (unless otherwise stated). With these results, we reached two important conclusions: 1) *ε*_c_ increases abruptly at the specific annealing temperature (*T*_s_), and 2) the *T*_s_ and the maximum *ε*_c_ increase as MW increases.

The P2 with the lowest MW (16 kg mol^−1^) showed poor stretchability, with no enhancement of the stretchability in the entire temperature range (Figure 2a; Figure S5, Supporting Information), because of the less‐entangled short polymer chains.^[^ ^31^, ^32^ ^]^ However, the P2 with increased MW showed significantly enhanced stretchability after the thermal annealing at temperatures higher than the *T*_s_. Although the P2 polymers with the MWs of 44 and 47 kg mol^−1^ showed a negligible difference in the MW, P2 (47 kg mol^−1^) exhibited a relatively high stretchability, presumably due to reinforced chain entanglements. Consequently, the P2 with the highest MW (47 kg mol^−1^) yielded a maximum *ε*_c_ of >200% (Figure 2a). To the best of our knowledge, this is the highest value reported for ISPSs.

Differential scanning calorimetry (DSC) results correlated the *T*_s_ and crystallization behavior of P2 (Figure 2b). The full DSC curves are shown in Figure S8a (Supporting Information). The DSC curve of P1 showed a single peak, indicating the crystallization temperature (*T*_c_) of ≈165 °C, which is similar to the optimal annealing temperature for the P1 thin films (Figure 1). In contrast, the DSC curves of the P2 polymers exhibited two distinct *T*_c_ peaks at temperatures higher than 200 °C, where P1 and P2 were stable at less than 400 °C (Figure S9, Supporting Information). More interestingly, the second *T*_c_ (*T*_c2_) observed at the relatively high temperature and the ratio of enthalpy change (△*H*) for *T*_c2_/*T*_c1_ peaks increased as the MW of P2 increased, where the *T*_c1_ peaks were likely to be associated with the growth of the face‐on crystallites (Figure S8b, Supporting Information). Since the *T*_c2_ peaks were observed for only P2, the *T*_c2_ peaks may be associated with the formation of the edge‐on crystallites. The high temperature and △*H* ratio for the *T*_c2_ peaks of the high‐MW P2 polymers can be ascribed to the relatively high thermal energy required for the structural phase transition of P2 with relatively long polymer chains.

The correlation between the *T*_c2_ temperature and the formation of the edge‐on crystallites was confirmed by the GIWAXD experiments on the P2 (47 kg mol^−1^) thin films annealed at various temperatures (25–340 °C) (Figure 2c; Figure S10 and Table S3, Supporting Information). The *L*_c_ of the face‐on crystallites for the (100) plane and the *π*–*π* stacking gradually increased until 260 and 240 °C, respectively, and decreased at higher temperatures. More importantly, the edge‐on crystallites were observed when the P2 thin films were annealed at temperatures higher than 260 °C. The *L*_c_ of the edge‐on crystallites was maximized at 280 °C. Since this temperature was almost identical to the *T*_s_ for P2 (47 kg mol^−1^), the suddenly increased edge‐on crystallite ratio (*R*_edge‐on_) at the same temperature (280 °C) showed simultaneously enhanced stretchability and crystallinity owing to the formation of the edge‐on crystallites (Figure 2c).

### 2.4 Dynamic Orientation of the Polymer Crystallites

**Figure 3:**
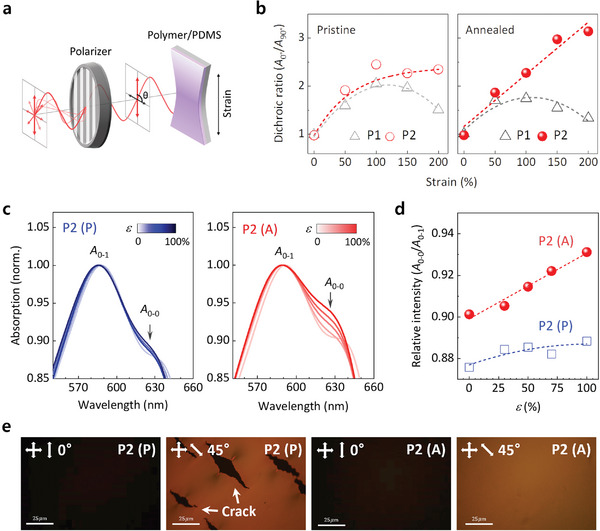
Dynamic orientation of polymer crystallites. a) Experimental setup for a polarized UV–vis absorption spectroscopy system consisting of a Glan–Taylor polarizer. b) DR (*A*_0°_/*A*_90°_) of the P1 and P2 thin films on the PDMS substrates at various strain values (*ε* = 0%, 50%, 100%, 150%, and 200%), where *A*_0°_ and *A*_90°_ indicate absorbance in the direction parallel and perpendicular to the stretching direction, respectively. c) Polarized UV–vis absorption spectra of the P2 thin films before (P) and after (A) the thermal annealing at various strain values (*ε* = 0%–100%); the *A*_0–0_ and *A*_0–1_ indicate absorption peaks for the 0–0 and 0–1 electronic transition modes, respectively. d) The relative intensity variation of the two distinct peaks (*A*_0–0_/*A*_0–1_) of the P2 thin films before (P) and after (A) thermal annealing, which were extracted from the UV–vis absorption spectra shown in c. e) POM images of the stretched (*ε* = 200%) P2 thin films before (P) and after (A) thermal annealing taken at sample angles of 0° and 45°, respectively, relative to the polarizer, where the polarizer and analyzer were set perpendicular to each other.

Polarized UV–vis absorption spectroscopy was used to investigate the effect of the polymer crystallites on the stretchability enhancement, where the absorption spectra were recorded at various polarization angles (**Figure** **3**a). The absorption intensity of the stretched thin films increased in the direction parallel (*θ* = 0°) to the tensile stretching direction and decreased as the polarization angle increased (*θ* = 0°→90°), showing that the polymer chains and crystallites were aligned in the direction parallel to the tensile stretching direction (Figure S11, Supporting Information). The dichroic ratio (DR, *A*_0°_/*A*_90°_) of the pristine P1 and P2 thin films increased as the tensile strain increased and eventually decreased or saturated at a strain higher than ≈100% (Figure 3b), which were their *ε*_c_ values. The saturated DR of the pristine P2 thin film was likely due to the relatively fewer generated cracks than for the pristine P1 thin film (Figures S4 and S5, Supporting Information). Further, the annealed P1 thin film showed a similar trend with the pristine P1 thin film. However, the annealed P2 thin film exhibited a linearly increased DR exceeding 3.0 at *ε* = 200%, showing that the polymer crystallites in the annealed P2 thin films were oriented and aligned under the applied tensile strain of up to *ε* = 200% without losing mechanical tension. This was attributed to enhanced stretchability.

We observed more interesting results when comparing the polarized UV–vis absorption spectra of the pristine and annealed P2 thin films obtained at *θ* = 0° under various tensile strains (Figure 3c). Contrary to the almost identical absorption spectra of the pristine P2 thin films, regardless of the applied tensile strains, the annealed P2 thin films exhibited gradually increased absorption intensities for the 0–0 mode transition when normalized to that for the 0–1 mode transition. Considering the edge‐on crystallites that existed in only the annealed P2 thin films, the gradually increased ratios of the absorption intensities (*A*_0–0_/*A*_0–1_) indicated dynamically oriented edge‐on crystallites in the direction parallel to the stretching direction under applied tensile strain (Figure 3d).^[^ ^33^ ^]^

The well‐oriented polymer crystallites in the annealed P2 thin films under tensile strain were verified using polarized optical microscopy (POM) results (Figure 3e). While the POM images taken at 0° were dark, the images taken at 45° exhibited a bright color, showing well‐oriented polymer crystallites in the P2 thin films under tensile strain.^[^ ^13^, ^34^, ^35^ ^]^ Compared with the pristine P2 thin film, the annealed P2 thin film showed a significantly brighter color in the image, without any cracks on the surface, indicating the optimal orientation of the polymer crystallites at the same applied tensile strain.

**Figure 4:**
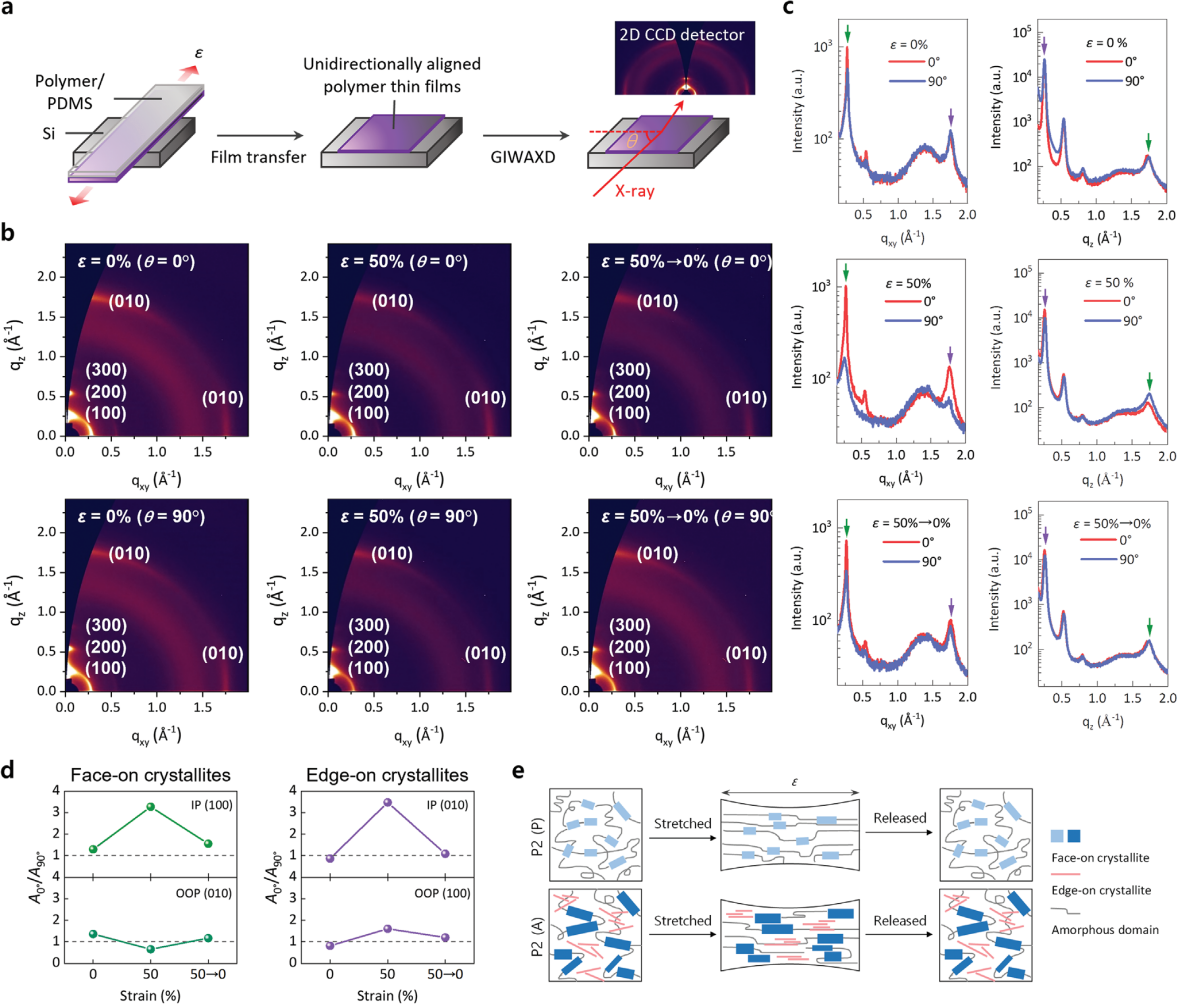
Direction‐dependent GIWAXD. a) Schematic illustration of the process for stretched thin film preparation on Si substrates. b) Reciprocal space maps for the annealed P2 thin films before and after stretching; the *ε* values of 0%, 50%, and 50%→0% indicate pristine, stretched (*ε* = 50%), and stretched/released (*ε* = 50%→0%) thin films, respectively, before transferring to the Si substrates. The reciprocal space maps were taken at X‐ray beam directions parallel (*θ* = 0°) and perpendicular (*θ* = 90°) to the film stretching (i.e., polymer orientation) direction. c) Corresponding linecut profiles of the annealed P2 thin films in the IP (left) and OOP (right) directions. The green and violet arrows indicate GIWAXD peaks representing face‐on and edge‐on crystallites, respectively. d) *A*_0°_/*A*_90°_ values for annealed P2 thin films under various stretching conditions indicating a dynamic orientation of the face‐on and edge‐on crystallites, where *A*_0°_ and *A*_90°_ indicate the peak areas obtained in the direction parallel and perpendicular to the stretching direction. e) Schematic representation of the dynamic orientation of long‐range‐ordered face‐on and edge‐on crystallites in the annealed P2 thin film (bottom) compared to short‐range‐ordered face‐on crystallites in the pristine P2 thin film (top).

Further, the dynamic orientation of the polymer crystallites under tensile strain was confirmed by the GIWAXD measurements with varying incidence directions of X‐ray beams. The pristine and annealed P2 thin films were transferred with or without tensile strain onto precleaned Si substrates by the dry‐contact transfer method.^[^ ^20^, ^36^ ^]^ The GIWAXD signals were recorded with X‐ray beam incidents in the direction parallel (*θ* = 0°) and perpendicular (*θ* = 90°) to the film stretching direction (**Figure** **4**a). To prepare the stretched thin films for the GIWAXD experiments, the annealed P2 thin films were stretched at *ε* = 50% and transferred onto the Si substrates. Other thin films were stretched and released to the original position (*ε* = 50%→0%) before transferring onto the Si substrates. Hence, their GIWAXD signals were recorded using X‐ray beam incidents at *θ* = 0° and *θ* = 90°, respectively, and compared with those of the annealed P2 thin films transferred without any mechanical strain (Figure 4b,c). For comparison, the pristine P1, annealed P1, and pristine P2 thin films were investigated using the same procedure (Figures S12–S14 and Tables S4 and S5 Supporting Information).

After the film transfer, the annealed P2 thin films showed bimodal crystalline structures identical to those of the spin‐cast thin films (Figure 1), confirming the effective film transfer process without disturbing the molecular orientation of the transferred thin films. The annealed P2 thin film transferred without mechanical strain showed an isotropic molecular configuration regardless of the X‐ray beam directions (Figure 4c), which is typical for non‐oriented polymer thin films.^[^ ^33^, ^37^ ^]^ In contrast, the annealed P2 thin film stretched at *ε* = 50% exhibited anisotropic molecular orientation in the IP direction. This anisotropy can be quantified by the significantly enhanced *A*_0°_/*A*_90°_ values, as the ratio of the peak area obtained at *θ* = 0° to that obtained at *θ* = 90°, approaching 4.0 at *ε* = 50% for the face‐on and edge‐on crystallites in the IP direction (Figure 4d). This is consistent with the results reported by DeLongchamp et al.^[^ ^33^ ^]^ The *A*_0°_/*A*_90°_ value of the annealed P2 thin film, stretched at *ε* = 50% and released to the original position before film transfer, was reduced to ≈1.0, which was almost identical to that of the unstretched thin film.^[^ ^20^ ^]^ These results indicate a fully recovered molecular orientation upon mechanical relaxation. Notably, the rDoC values of the corresponding P2 thin films were almost unchanged (Figure S15, Supporting Information), which corresponds with previous reports.^[^ ^12^, ^38^ ^]^ Additionally, there were no noticeable changes in the *A*_0°_/*A*_90°_ values in the OOP direction because of the compressive force applied in the vertical direction with respect to the film stretching direction upon the tensile stretching of the thin films.^[^ ^20^ ^]^

The direction‐dependent GIWAXD results showed the dynamic and unidirectional orientation of the polymer crystallites in the annealed P2 thin film parallel to the stretching direction. The orientation of the face‐on crystallites in the lateral direction cannot improve the intermolecular interaction because of the steric hindrance by alkyl side chains. Thus, dynamically oriented edge‐on crystallites and the associated reinforcement of noncovalent interactions under tensile strain can be mainly responsible for the stretchability enhancement of the annealed P2 thin films. This may be explained by the previous results reported by Bao et al.^[^ ^20^ ^]^ More specifically, long‐range‐ordered edge‐on crystallite domains with strengthened interchain noncovalent interactions can act like pseudo‐cross‐linking sites covalently holding adjacent amorphous regions, thereby inducing the concurrently improved crystallinity and stretchability of the annealed P2 thin films (Figure 4e).^[^ ^9^ ^]^ This can be explained by relatively high and nearly unchanged *L*_c, *π*–*π*_ of edge‐on crystallites in annealed P2 thin films under tensile strain compared to that of face‐on crystallites (Table S5, Supporting Information). Considering the simulation results reported by Barrat et al. showing that the crystalline regions withstand the force applied by tensile strain at an earlier stage better than the amorphous regions,^[^ ^39^ ^]^ our crystalline structure‐based model suggested for simultaneously enhanced crystallinity and stretchability may be reasonable.

### 2.5 Anisotropic Charge Transport and Mechanical Robustness

**Figure 5:**
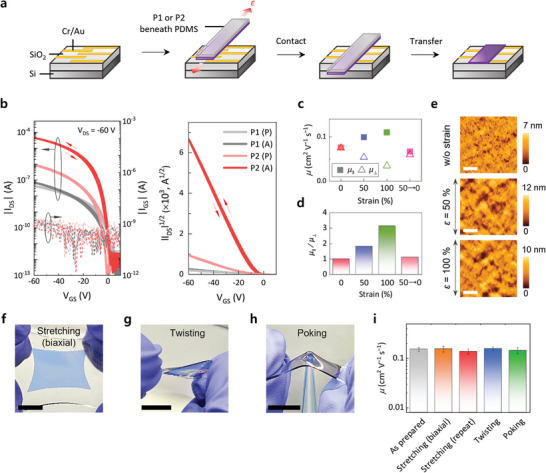
Anisotropic charge transport and mechanical robustness of stretched P2 thin films. a) Schematic representation for the device fabrication process based on stretched P2 thin films. b) Transfer characteristics of the OFETs fabricated with spin‐cast P1 and P2 thin films before (P) and after (A) thermal annealing. c) Hole mobility variation of the annealed P2 thin films obtained at various strain values (*ε* = 0%, 50%, 100%, and 50%→0%) in the direction parallel (*μ*_∥_) and perpendicular (*μ*_⊥_) to the stretching direction. d) *μ*_∥_/*μ*_⊥_variations of the devices based on the annealed P2 thin films transferred under various stretching conditions (*ε* = 0%, 50%, 100%, and 50%→0%). e) AFM height images of the annealed P2 thin films stretched at various strain values (*ε* = 0%, 50%, and 100%). Scale bar: 500 nm. f–h) Photographs of annealed P2 thin films on the PDMS substrates, where the films were stretched f) stretched biaxially, g) twisted, and h) poked by a pipette tip. Scale bar: 1 cm. i) Hole mobilities of the annealed P2 thin films released from the various stretching conditions. The repeated stretching condition can be found in Movie S1 (Supporting Information).

Organic field‐effect transistors (OFETs) were fabricated using spin‐cast P1 and P2 thin films to investigate the effect of crystallinity and crystalline structures on their electrical properties. Further, we investigated the charge transport properties of the annealed P2 thin films under various stretching conditions by transferring the annealed P2 thin films onto SiO_2_ substrates (**Figure** **5**a).^[^ ^36^ ^]^ For the spin‐cast thin films, the optimal annealing temperatures (150 and 270 °C for P1 and P2, respectively) were determined by calculating the highest *µ* value among the values obtained at various annealing temperatures (Figure S16, Supporting Information). The detailed device parameters are summarized in Figure S17 and Table S6 (Supporting Information). It is observed that the annealed P2 devices yield the highest *µ* values (0.06–0.19 cm^2^ V^−1^ s^−1^), which are approximately two orders of magnitude higher than those of the pristine P2 devices (Figure 5b). Further, these values are three orders of magnitude higher than those of the optimized P1 devices annealed at 150 °C. Additionally, the annealed P2 devices showed a remarkably high on/off ratio (>10^8^), low threshold voltage (*V*_T_ <−8 V), and low deep trap density (<10^12^ eV^−1^ cm^−2^) (Figure S18 and Table S6, Supporting Information). Furthermore, they showed ideal transistor characteristics with nearly monotonic *µ* in the entire saturation regime (i.e., applied gate bias (*V*_GS_) = −20 to −60 V) (Figure S18c, Supporting Information). These excellent device performances can be ascribed to the reduced structural disorders induced by the improved thin film crystallinity and enhanced edge‐on crystallite ratio, which are favorable for the increased *µ* of polymer semiconductors (Figure 1 and 2).^[^ ^40^, ^41^, ^42^ ^]^

The annealed P2 thin films maintained these excellent electrical properties after tensile stretching, showing high *µ* values under various tensile strains (*ε* = 0%–100%) (Figure 5c). More importantly, the stretched P2 thin films showed anisotropic charge transport (Figure 5c,d), inducing *μ*_∥_/*μ*_⊥_ values, as the ratio of the *µ* values in the direction parallel (*μ*_∥_) and perpendicular (*μ*_⊥_) to the charge transport direction gradually increased to 3.0 as the tensile strain increased to 100%. Next, the ratio was released to that of the unstretched thin film (*μ*_∥_/*μ*_⊥_ ≈ 1) upon relaxation, corroborating the GIWAXD results (Figure 4d). Atomic force microscopy (AFM) results showed gradually enlarged grain sizes for the annealed P2 thin films as the tensile strain increased because of the orientation of the polymer crystallites, and accordingly, the *L*_c_ increased upon stretching (Figure 5e). This result corresponds with the GIWAXD results (Figure 2 and 4).

Finally, the annealed P2 thin films showed excellent mechanical robustness, indicated by the high *µ* obtained even after stretching under harsh conditions (Figure 5f–i; Figure S19 and Movie S1, Supporting Information). The annealed P2 thin films, which were stretched biaxially (Figure 5f), repeatedly stretched (Movie S1, Supporting Information), twisted (Figure 5g), and poked by a pipette tip (Figure 5h), exhibited average *µ* values over 0.1 cm^2^ V^−1^ s^−1^, which were similar to those of the as‐prepared (unstretched) thin films (Figure 5i). These results show the wide applicability of the annealed P2 thin films with simultaneously improved stretchability and *µ* in a variety of high‐performance and reliable stretchable electronics.

## 3 Conclusion

The fluorinated polymer semiconductor reported in this work shows, for the first time, simultaneously improved crystallinity and stretchability enabled by the thermally‐assisted tailoring of the crystalline structure. This polymer allows a deep understanding of the crystallinity–stretchability relationship for stretchable polymer semiconductors and provides a design guideline for highly stretchable semi‐crystalline polymer semiconductors. This design strategy can broaden the availability of thousands of donor and acceptor moieties already developed for highly crystalline polymer semiconductors, thereby accelerating the research and development of next‐generation stretchable electronics.

## 4 Experimental Section

### UV–Vis Absorption Spectroscopy

UV–vis absorption spectra were obtained using a commercial UV–vis–near‐IR spectrophotometer (V‐770, Jasco Inc.). The absorption spectra of the P1 and P2 solutions and thin films were recorded at 25 °C. For polarized UV–vis absorption spectroscopy, the polymer thin films on the polydimethylsiloxane (PDMS) substrates were stretched at various strains and mounted in the spectrophotometer. Subsequently, the polarized UV–vis absorption spectra of the thin films were obtained using the polarized light generated by passing a monochromic light through a Glan–Taylor polarizer (Edmund Optics Ltd.).

### GIWAXD

GIWAXD measurements were performed using a 9A U‐SAXS beamline of the Pohang Accelerator Laboratory in Korea. The X‐rays from the in‐vacuum undulator were monochromated (*E* = 11.08 keV), with an incidence angle of 0.12°. GIWAXD patterns were obtained using a 2D couple‐charged device detector (MX170‐HS; Rayonix Inc.). The detector was located ≈210 mm from the center of the sample. The *R*_edge‐on_ for each film was calculated using the following equations:^[^ ^41^, ^43^ ^]^

(1) $$R_{\mathrm{edge}-\mathrm{on},\;\pi -\pi \;\mathrm{stac}\mathrm{king}}=\frac{I\;{\left(\chi \right)}_{0-45^{\circ }}}{I\;{\left(\chi \right)}_{0-45^{\circ }}+I\;{\left(\chi \right)}_{45-90^{\circ }}}$$

(2) $$R_{\mathrm{edge}-\mathrm{on},\;(100)\;\mathrm{plane}}=\frac{I\;{\left(\chi \right)}_{45-90^{\circ }}}{I\;{\left(\chi \right)}_{0-45^{\circ }}+I\;{\left(\chi \right)}_{45-90^{\circ }}}$$

where *I*(*χ*) indicate the peak intensity at the assigned azimuthal angles. For direction‐dependent GIWAXD experiments, GIWAXD patterns were first obtained in the direction parallel to the stretching direction (*θ* = 0°). Thereafter, the samples were rotated by 90° for an additional measurement at *θ* = 90°. The rDoC values were determined by using the previously reported method.^[^ ^43^, ^44^ ^]^

### Crack Onset Strain

The PDMS freestanding films were prepared by mixing a PDMS base (SYLGARD^®^ 184A, Dow Corning Co.) and a cross‐linker (SYLGARD^®^ 184B, Dow Corning Co.) at a ratio of 10:1 by weight.^[^ ^45^ ^]^ The mixture was poured into a Petri dish and cured for over 24 h at 25 °C. The P1 and P2 thin films were spun cast from the toluene solutions at a concentration of 10 mg mL^−1^ onto SiO_2_ substrates passivated with *n*‐decyltrichlorosilane (*n*‐DTS, Gelest Inc.). The P1 and P2 thin films before and after the thermal annealing were transferred onto the PDMS substrates at 150 °C in ambient air. Next, the thin films stretched at various tensile strains were mounted onto glass slides, and the OM images of the stretched P1 and P2 thin films were captured using an optical microscope (ECLIPSE LV100N POL, Nikon Inc.) to determine the *ε*_c_ values.

### FOE

The elastic moduli and yield points of the pristine and annealed P2 thin films were determined using the following equation:^[^ ^46^ ^]^

(3) $$E_{f}=3E_{s}\left(\frac{1-v_{f}^{2}}{1-v_{s}^{2}}\right){\left(\frac{d}{2\pi h}\right)}^{3}$$

where *E*_f_ and *E*_s_ are the elastic moduli of the film and substrate, respectively, *v*_f_ and versus are the Poisson's ratios of the polymer semiconductor film (≈0.35) and PDMS substrate (≈0.5), respectively, *d* is the wavelength of the buckles, and *h* is the thickness of the film.

### NI

For the NI experiments, the P1 and P2 thin films (thickness of ≈1 µm) were prepared by drop‐casting toluene solutions on glass substrates. Next, the films were mounted in a nanoindenter (Ultra Nanoindentation Tester, Anton Paar GmbH Ltd.) and pressed by the Berkovich tip. The elastic moduli were determined using the Oliver and Pharr method with a Poisson's ratio of 0.4 for the P1 and P2 films.^[^ ^47^ ^]^

### DSC

DSC experiments were performed using a DSC system (TGA55, TA Instruments Inc.) at heating and cooling rates of 10 °C min^−1^ in the temperature range of 27–350°C in a nitrogen atmosphere.

### POM

POM images were recorded using a polarizing microscope (ECLIPSE LV100N POL, Nikon Inc.) with a polarizer and analyzer set perpendicular to each other. The images were taken at sample angles of 0° and 45° relative to the polarizer.

### OFET

For the OFETs prepared using spin casting, the Ni (5 nm)/Au (50 nm) source and drain electrodes were patterned on SiO_2_ (300 nm)/n^++^Si (500 µm) substrates through a conventional photolithography process. The channel width and length were 1000 and 20 µm, respectively. All metal electrodes were deposited by e‐beam evaporation at 7 × 10^−7^ Torr. Thereafter, the SiO_2_ substrates were cleaned by a sequential sonication process using acetone and isopropyl alcohol. The substrates were dried in an oven overnight at >100 °C. Next, the substrates were passivated with *n*‐DTS in a toluene solution (10 µL mL^−1^), followed by UV–ozone treatment for 10 min in ambient air. After transferring the *n*‐DTS‐passivated SiO_2_ substrates into a nitrogen‐filled glove box, the P1 and P2 thin films were spun cast from toluene solutions with a concentration of 5 mg mL^−1^ on top of the substrates. For the OFETs fabricated with stretched thin films, the annealed P2 thin films were transferred onto the PDMS substrates using the dry‐contact transfer method.^[^ ^36^ ^]^ Subsequently, the thin films were transferred onto the *n*‐DTS‐passivated SiO_2_ substrates with a pre‐patterned Cr (5 nm)/Au (50 nm) source and drain electrodes for completing the device fabrication. Note that all the transferred P2 thin films were annealed at 150 °C in a nitrogen‐filled glove box to promote good adhesion between the P2 thin films and the SiO_2_ substrates. The device parameters were characterized using a semiconductor parameter analyzer (4200A‐SCS, Keithley Inc.) in a nitrogen‐filled glove box.

### AFM

The height and phase images of the P1 and P2 thin films were obtained using a tapping‐mode AFM (AFM5100N, Hitachi, Ltd.). The root‐mean‐square values were determined using built‐in software.

## Conflict of Interest

The authors declare no conflict of interest.

## Supporting information

Supporting Information

Supplemental Movie 1

## Data Availability

The data that support the findings of this study are available from the corresponding author upon reasonable request.
