# Supplementary material for: A Semi‐Crystalline Polymer Semiconductor with Thin Film Stretchability Exceeding 200%
Source: Adv Sci (Weinh). 2023 May 25;10(22):2302683. doi: 10.1002/advs.202302683 (PMC10401152; doi:10.1002/advs.202302683)
Supplement: Supplementary file 1 — Supporting Information [file ADVS-10-2302683-s001.pdf]

ADVANCED SCIENCE
Open Access

Supporting Information

for *Adv. Sci.*, DOI 10.1002/advs.202302683

A Semi-Crystalline Polymer Semiconductor with Thin Film Stretchability Exceeding 200%

*Yejin Kim, Hyungju Ahn, Dahyeon Yoo, Mingi Sung, Hyeonjin Yoo, Sohee Park, Junghoon Lee and Byoung Hoon Lee**

Supporting Information

A semi-crystalline polymer semiconductor with thin film stretchability exceeding 200%

*Yejin Kim, Hyungju Ahn, Dahyeon Yoo, Mingi Sung, Hyeonjin Yoo, Sohee Park, Junghoon Lee, and Byoung Hoon Lee**

Table of Contents

Note 2

Scheme S1 4

Tables S1–S6 5–10

Figures S1–S19 11–29

Materials

(4,4-dihexadecyl-4H-cyclopenta[1,2-b:5,4-b']dithiophene-2,6-diyl)bis(trimethylstannane) (M1) was purchased from 1-Materials Inc., and 4,7-bis(5-bromothiophen-2-yl)-2-octyl-2H-benzo[d][1,2,3] triazole (M2) and 4,7-bis(5-bromothiophen-2-yl)-5,6-difluoro-2-octyl-2H-benzo[d][1,2,3]triazole (M3) were obtained from Suna Tech Inc. All other chemicals and reagents were purchased from Sigma-Aldrich, TCI, and Alfa Aesar and used without any further purification. All solvents are ACS grade, unless otherwise noted.

Synthesis and characterization

(4,4-dihexadecyl-4H-cyclopenta[1,2-b:5,4-b']dithiophene-2,6-diyl)bis(trimethylstannane) (M1) (0.21 mmol), 4,7-bis(5-bromothiophen-2-yl)-2-octyl-2H-benzo[d][1,2,3] triazole (M2) or 4,7-bis(5-bromothiophen-2-yl)-5,6-difluoro-2-octyl-2H-benzo[d][1,2,3]triazole (M3) (0.21 mmol), bis(dibenzylideneacetone)palladium(0) (6.3 μmol), and tri(*o*-tolyl)phosphine (25.19 μmol) in anhydrous *o*-xylene (4 mL) were added to a Schlenk flask and purged with argon for 10 min. Subsequently, the reaction mixture was heated to 140°C for 12–72 h. After cooling to room temperature, the solution was poured into methanol (300 mL). The precipitate was filtered off and subjected to sequential Soxhlet extraction with methanol (24 h), acetone (24 h), and hexane (24 h) to remove the low-molecular-weight fractions. The residue was extracted with chloroform (CF) to produce brown P1 and P2 after precipitating again from methanol and drying *in vacuo*.

Poly(4-(5-(4,4-dihexadecyl-4H-cyclopenta[1,2-b:5,4-b']dithiophen-2-yl)thiophen-2-yl)-2-octyl-7-(thiophen-2-yl)-2H-benzo[d][1,2,3]triazole) (PCDTBTA, P1)

Isolated yield = 63%. Number-average molecular weight (MW) = 24 and 43 kg mol^{-1}, weight-average MW = 43 and 90 kg mol^{-1}, and polydispersity index (PDI) = 2.0 and 2.1. Proton nuclear magnetic resonance (^{1}H NMR) ($CDCl_3$, 400 MHz, 298 K): δ ppm 8.19–7.33 (br, 6H), 7.17–6.94 (br, 2H), 4.97–4.78 (br, 2 H), 2.30–2.18(br, 2H), 2.04–1.80 (br, 4H), 1.61–0.94 (br, 66H), 0.92–0.77 (br, 9H). Anal. Calcd. for $C_{63}H_{93}N_3S_4$: C, 74.13; H, 9.18; N, 4.12; S, 12.57.

Poly(4-(5-(4,4-dihexadecyl-4H-cyclopenta[1,2-b:5,4-b']dithiophen-2-yl)thiophen-2-yl)-5,6-difluoro-2-octyl-7-(thiophen-2-yl)-2H-benzo[d][1,2,3]triazole) (PCDTFBTA, P2)

Isolated yield = 62%. Number-average MW = 16, 25, 44, and 47 kg mol^{-1}, weight-average MW = 30, 57, 92, and 94 kg mol^{-1}, PDI = 1.9, 2.3, 2.1, and 2.0. ^{1}H NMR ($CDCl_3$, 400 MHz, 298 K): δ ppm 8.41–7.50 (br, 4H), 7.13–6.84 (br, 2H), 5.02–4.60 (br, 2H), 2.41–2.16 (br, 2H), 1.95–1.71 (br, 4H), 1.67–1.07 (br, 66H), 0.93–0.79 (br, 9H). Anal. Calcd. for $C_{63}H_{91}F_2N_3S_4$: C, 71.61; H, 8.68; F, 3.60; N, 3.98; S, 12.14.

Methods

^{1}H NMR

^{1}H NMR spectra were recorded on a VNMRS 600 (Varian, USA) spectrophotometer using $CDCl_3$ and $C_2D_2Cl_4$ as solvents and tetramethylsilane (TMS) as the internal standard, and matrix-assisted laser desorption/ionization mass spectrometry spectra were obtained from Ultraflex III (Bruker, Germany).

High-temperature gel permeation chromatography

The number-average MWs, weight-average MWs, and PDI values of the polymer products were determined by high-temperature gel permeation chromatography measurements using PL-GPC 220 (Agilent Inc.) based on a series of monodisperse polystyrene standards in a trichlorobenzene (TCB) eluent. The experiments were performed on polymer TCB solutions with a concentration of 2 mg mL^{-1} at 150°C. The flow rate was 1 mL min^{-1}.

Scanning electron microscopy

Cross-sectional scanning electron microscopy (SEM) images were obtained using a field-emission SEM (S-4800, Hitachi Inc.) operated at an accelerating voltage of 15 kV.

Thermogravimetric analysis

The thermogravimetric analysis (TGA) curves of P1 and P2 were obtained using a commercial TGA system (Q600, TA Instruments Inc.). The measurements were carried out in a nitrogen atmosphere with a heating rate of 10°C min^{-1} in the temperature range of 30°C–600°C.

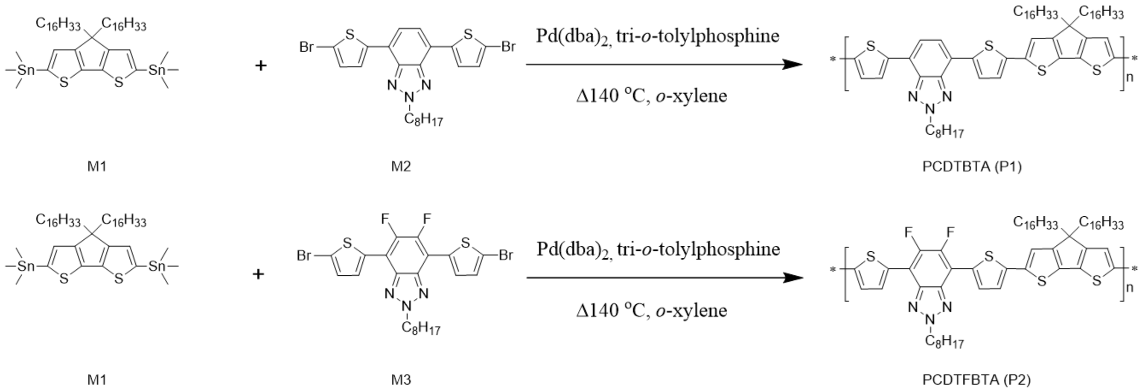

Scheme S1. Synthesis routes of P1 and P2.

Table S1. Crystallographic parameters of the pristine and annealed P1 and P2 thin films

Polymer	Annealing temperature (°C)	In-plane (IP, Å)				Out-of-plane (OOP, Å)			
		d_{100}	$L_{c,\ 100}$	$d_{\pi-\pi}$	$L_{c,\ \pi-\pi}$	d_{100}	$L_{c,\ 100}$	$d_{\pi-\pi}$	$L_{c,\ \pi-\pi}$
P1	-	27.65	59.54	-	-	25.61	55.29	-	-
	150	27.78	61.79	-	-	29.09	84.39	-	-
P2	-	24.99	49.88	3.65	21.26	24.35	52.53	3.67	20.22
	270	24.45	60.89	3.56	90.67	24.91	108.56	3.68	24.86

Table S2. Crystallographic parameters of the pristine and annealed P2 thin films spin-cast from CF and chlorobenzene (CB) solutions

Solvent	Annealing temperature (°C)	IP (Å)				OOP (Å)			
		d_{100}	$L_{c,\ 100}$	$d_{\pi-\pi}$	$L_{c,\ \pi-\pi}$	d_{100}	$L_{c,\ 100}$	$d_{\pi-\pi}$	$L_{c,\ \pi-\pi}$
CF	-	24.31	234.35	3.56	62.33	25.01	174.00	3.62	55.76
	270	24.69	44.59	3.69	19.73	-	-	3.66	19.94
CB	-	24.21	248.57	3.56	61.47	25.02	176.83	3.63	47.28
	270	24.48	46.46	3.62	25.55	-	-	3.65	20.64

Table S3. Crystallographic parameters of the P2 thin films before and after thermal annealing at various annealing temperatures

Annealing temperature (°C)	IP (Å)				OOP (Å)			
	d_{100}	$L_{c,\,100}$	$d_{\pi\text{–}\pi}$	$L_{c,\,\pi\text{–}\pi}$	d_{100}	$L_{c,\,100}$	$d_{\pi\text{–}\pi}$	$L_{c,\,\pi\text{–}\pi}$
-	24.84	52.61	-	-	-	-	3.65	29.64
200	24.38	141.34	-	-	-	-	3.63	50.02
220	24.40	184.20	-	-	-	-	3.63	60.73
240	24.32	227.74	-	-	-	-	3.61	71.88
260	24.22	266.24	3.57	91.86	25.27	139.90	3.62	62.83
280	24.12	192.93	3.57	97.65	24.99	253.92	3.61	53.60
300	24.53	80.11	3.58	92.40	25.00	221.24	3.64	34.75

Table S4. Crystallographic parameters of the pristine and annealed P1 thin films under various stretching conditions

Sample	Strain (%)	IP (Å)				OOP (Å)			
		d_{100}	$L_{c,\,100}$	$d_{\pi\text{–}\pi}$	$L_{c,\,\pi\text{–}\pi}$	d_{100}	$L_{c,\,100}$	$d_{\pi\text{–}\pi}$	$L_{c,\,\pi\text{–}\pi}$
Pristine (θ = 90°)	-	27.21	66.40	3.58	28.71	-	-	-	-
	30	27.57	61.69	3.58	30.30	-	-	-	-
	30→0	27.09	63.22	3.59	25.07	-	-	-	-
Pristine (θ = 0°)	-	27.00	63.30	3.55	25.82	-	-	-	-
	30	26.94	60.47	3.58	30.56	-	-	-	-
	30→0	27.00	62.73	-	-	-	-	-	-
Annealed (θ = 90°)	-	26.36	93.25	3.59	27.50	26.74	234.74	-	-
	30	26.62	91.02	3.60	27.81	26.98	201.89	-	-
	30→0	26.57	87.65	3.59	28.53	27.25	230.25	-	-
Annealed (θ = 0°)	-	26.41	87.46	3.61	26.14	26.73	219.78	-	-
	30	26.31	83.93	3.58	29.71	26.67	194.66	-	-
	30→0	26.53	82.00	3.60	26.06	26.74	206.68	-	-

Table S5. Crystallographic parameters of the pristine and annealed P2 thin films under various stretching conditions

Sample	Strain (%)	IP (Å)				OOP (Å)			
		d_{100}	$L_{c,\ 100}$	$d_{\pi\text{–}\pi}$	$L_{c,\ \pi\text{–}\pi}$	d_{100}	$L_{c,\ 100}$	$d_{\pi\text{–}\pi}$	$L_{c,\ \pi\text{–}\pi}$
Pristine (θ = 90°)	-	24.68	37.42	-	-	-	-	3.65	28.38
	50	25.79	30.87	-	-	-	-	3.67	29.71
	50→0	25.16	33.94	-	-	-	-	3.67	29.23
Pristine (θ = 0°)	-	24.93	38.72	-	-	-	-	3.66	28.23
	50	24.40	47.27	-	-	-	-	3.68	27.78
	50→0	24.73	39.67	-	-	-	-	3.67	27.77
Annealed (θ = 90°)	-	23.74	90.43	3.57	74.19	23.73	194.59	3.60	61.59
	50	24.82	36.01	3.57	72.04	23.97	168.15	3.61	41.92
	50→0	24.19	69.26	3.58	64.22	24.12	158.18	3.63	53.92
Annealed (θ = 0°)	-	23.29	273.71	3.57	70.86	23.79	183.24	3.63	54.80
	50	23.02	295.91	3.55	64.16	23.89	164.48	3.61	45.17
	50→0	23.49	271.09	3.57	66.25	24.12	155.82	3.64	41.10

Table S6. Device parameters of organic field-effect transistors fabricated with the P1 and P2 thin films

Polymer	Annealing temperature (°C)	μ_{max} ($cm^2\ V^{-1}\ s^{-1}$)	$\mu_{ave}{}^a$ ($cm^2\ V^{-1}\ s^{-1}$)	V_T (V)	ON/OFF ratio	SS ($V\ decade^{-1}$)	N_{tr} ($eV^{-1}\ cm^{-2}$)
P1[b]	-	7.2×10^{-5}	6.3×10^{-5}	−1.6	9.0×10^4	2.48	2.86×10^{12}
	150	4.1×10^{-4}	3.3×10^{-4}	−11.7	4.5×10^5	1.55	1.76×10^{12}
P2[b]	-	2.5×10^{-3}	2.1×10^{-3}	−13.5	5.4×10^6	0.81	8.89×10^{11}
	270	6.5×10^{-2}	4.1×10^{-2}	−8.0	2.5×10^8	0.82	9.01×10^{11}

[a]The μ_{ave} values were obtained from 10 independent devices, where the numbers in parentheses indicate standard deviations.
[b]The devices were fabricated with the P1 and P2 polymers with number-average MWs of 24 and 25 kg mol^{-1}, respectively.

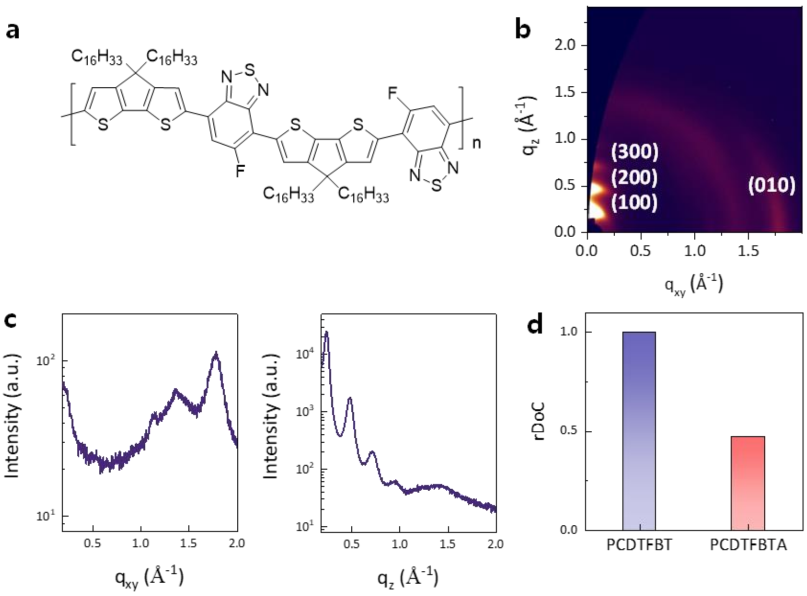

Figure S1. a, Chemical structure of the PCDTFBT. **b–c**, Two-dimensional grazing incidence wide-angle X-ray diffraction (GIWAXD) (b) reciprocal space map and (c) linecut profiles for a PCDTFBT thin film annealed at 200°C. **d**, Relative degree of crystallinity (rDoC) values for the PCDTFBT and PCDTFBTA (P2 in this study) thin films annealed at 200°C and 270°C, respectively.

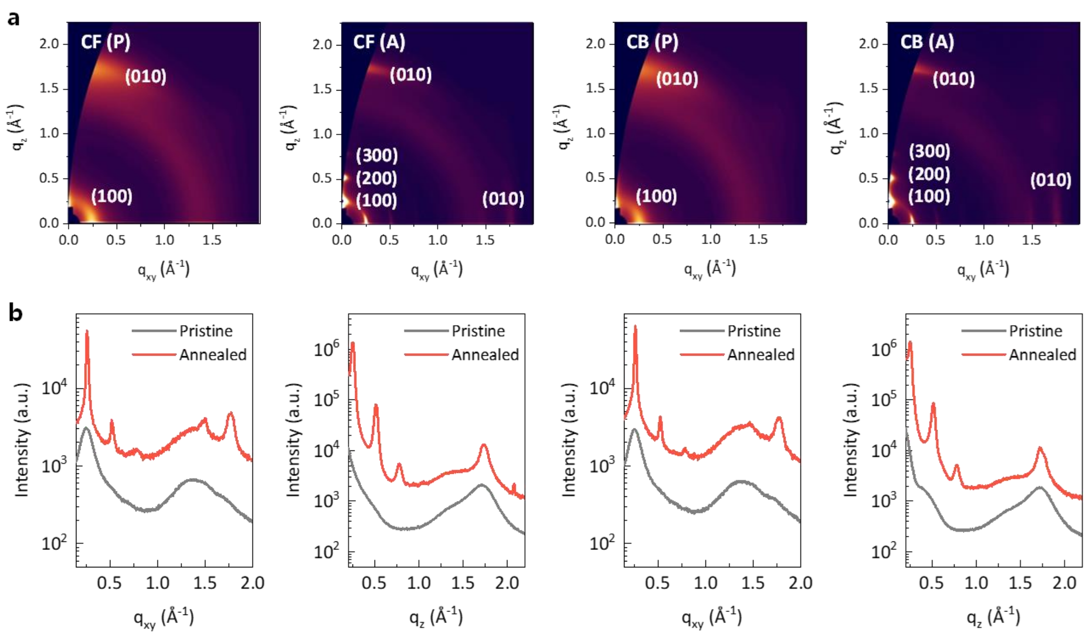

Figure S2. a–b, GIWAXD (a) reciprocal space maps and (b) linecut profiles for the P2 thin films spin-cast from CF and CB solutions before (pristine; P) and after (annealed; A) thermal annealing at 270°C.

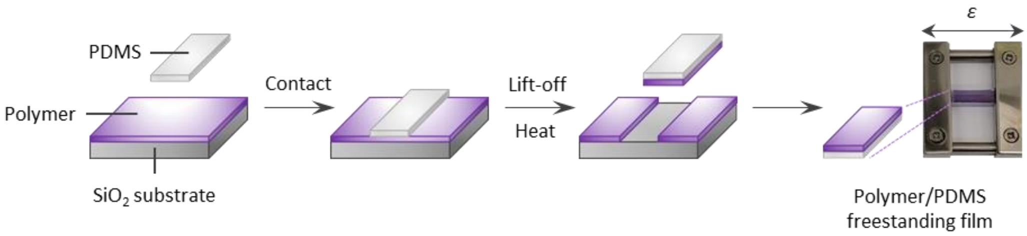

Figure S3. Schematic representation of the preparation of polymer semiconductor thin films on polydimethylsiloxane (PDMS) substrates.

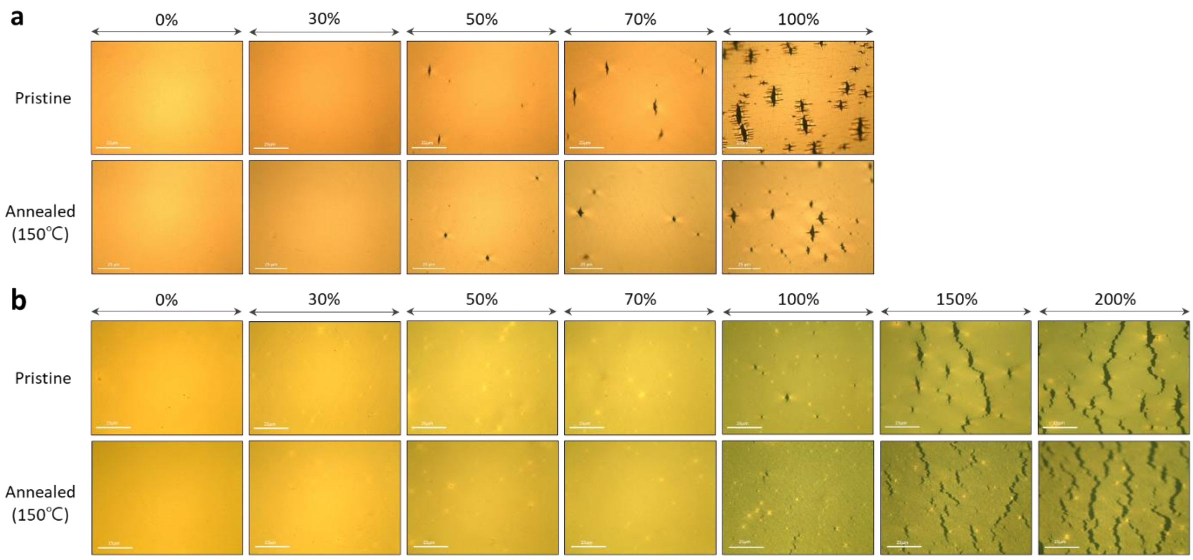

Figure S4. **a–b**, Optical microscopy (OM) images of the pristine and annealed P1 thin films with number-average MWs of (a) 24 kg mol^{-1} and (b) 43 kg mol^{-1} stretched at various strains. The thin films were prepared before and after thermal annealing at 150°C before transferring onto PDMS substrates. Scale bar: 25 μm.

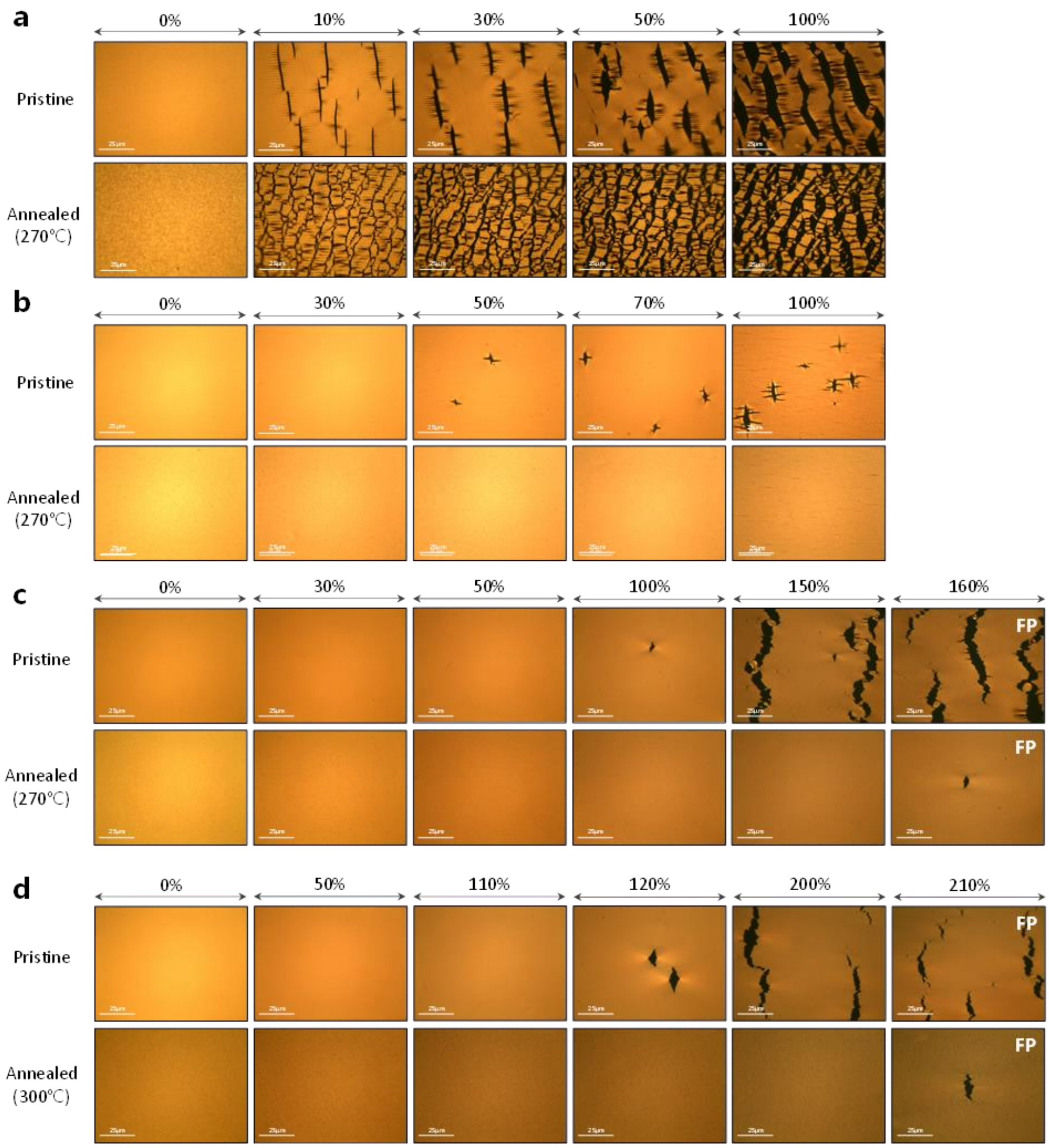

Figure S5. a–d, OM images of the pristine and annealed P2 thin films with number-average MWs of (a) 16 kg mol^{-1}, (b) 25 kg mol^{-1}, (c) 44 kg mol^{-1}, and (d) 47 kg mol^{-1} stretched at various strains. The thin films were prepared before and after thermal annealing at various temperatures (270°C for P2 with MWs of 16, 25, and 44 kg mol^{-1}; 300°C for P2 with a MW of 47 kg mol^{-1}) before transferring onto PDMS substrates. Scale bar: 25 μm.

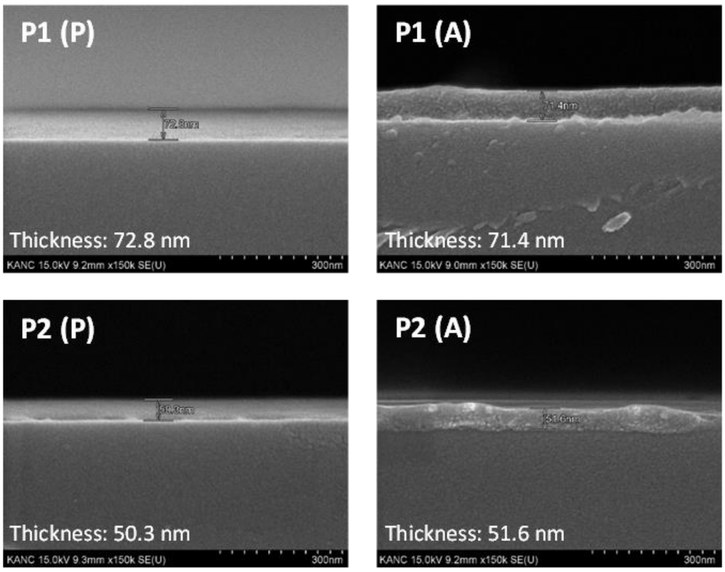

Figure S6. Cross-sectional scanning electron microscopy images of the pristine (P) and annealed (A) P1 and P2 thin films, where the numbers indicate the film thickness of the thin films. Scale bar: 300 nm.

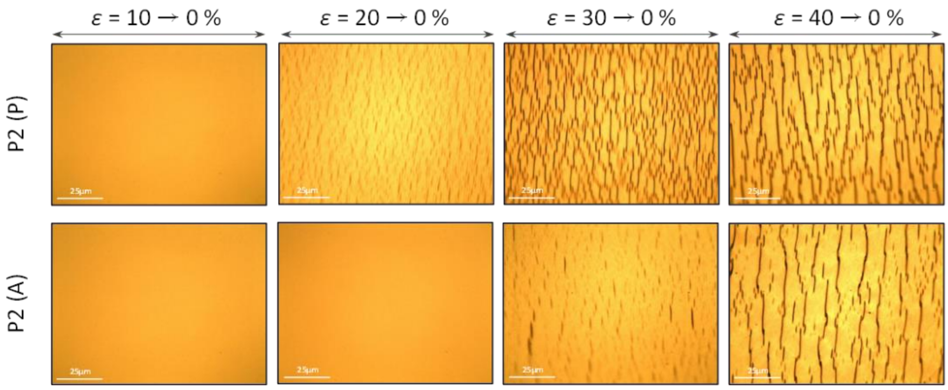

Figure S7. OM images of the pristine (P) and annealed (A) P2 thin films stretched at various strains (ε = 10%, 20%, 30%, and 40%) and subsequently released to the original position (ε = 0%). Scale bar: 25 μm.

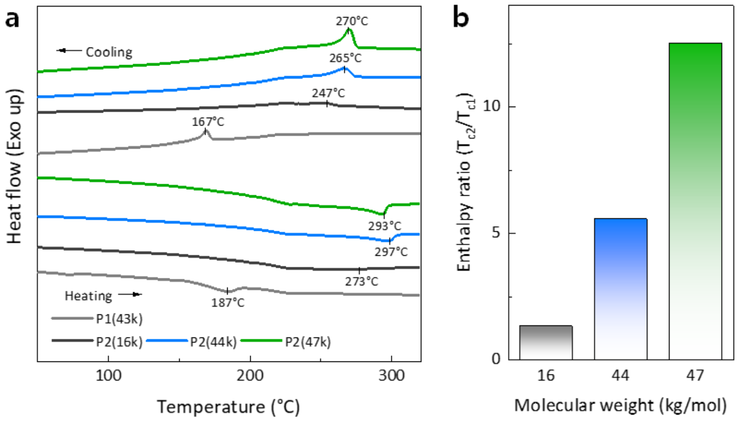

Figure S8. a, Differential scanning calorimetry curves of the P1 and P2 powders with various number-average MWs (43 kg mol^{-1} for P1; 16, 44, and 47 kg mol^{-1} for P2). **b**, Variations in the enthalpy ratios for T_{c2} peaks relative to T_{c1} peaks of P2 with various number-average MWs.

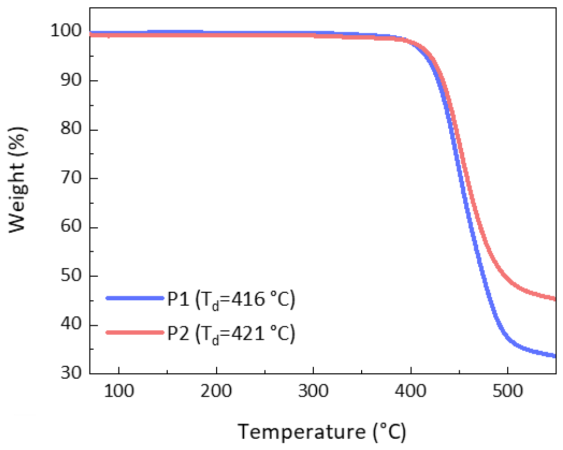

Figure S9. TGA curves of the P1 and P2 powders. The decomposition temperature (T_d) of each polymer was determined to be the temperature at 5% weight loss.

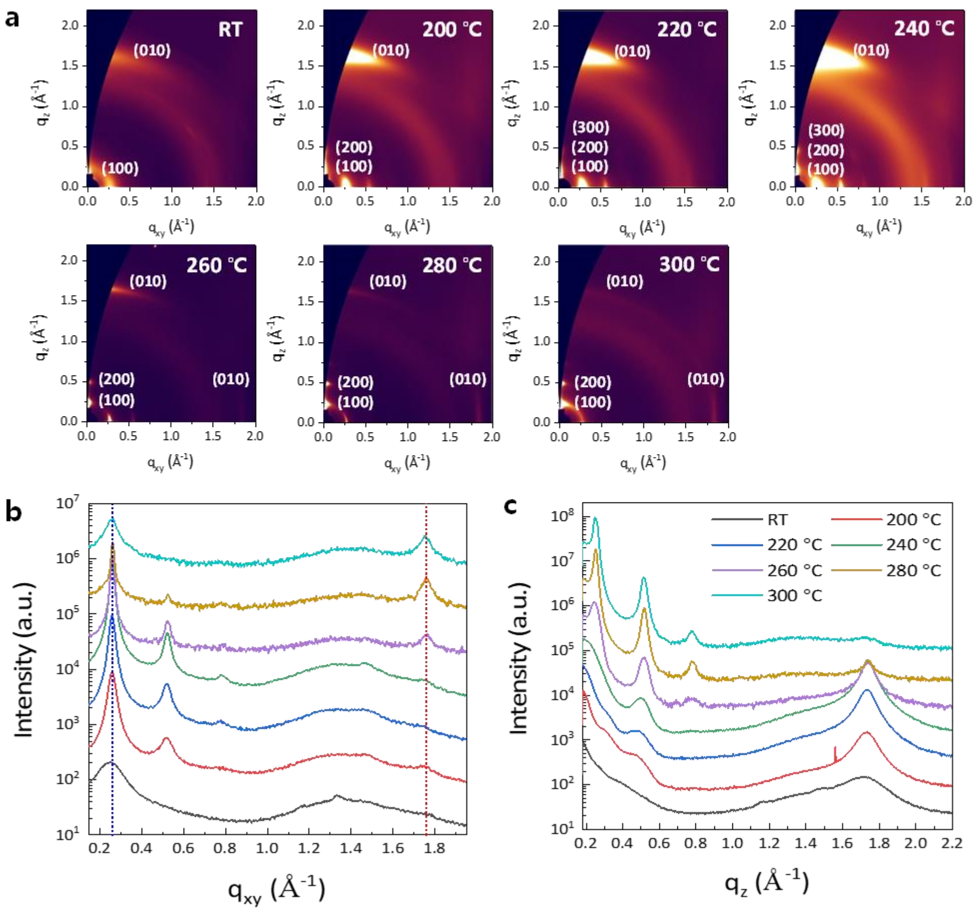

Figure S10. a, GIWAXD reciprocal space maps for the P2 thin films before (i.e., at room temperature (RT)) and after thermal annealing at various temperatures. **b–c**, Linecut profiles in (b) IP and (c) OOP directions extracted from the reciprocal space maps for P2 thin films before and after thermal annealing at various temperatures.

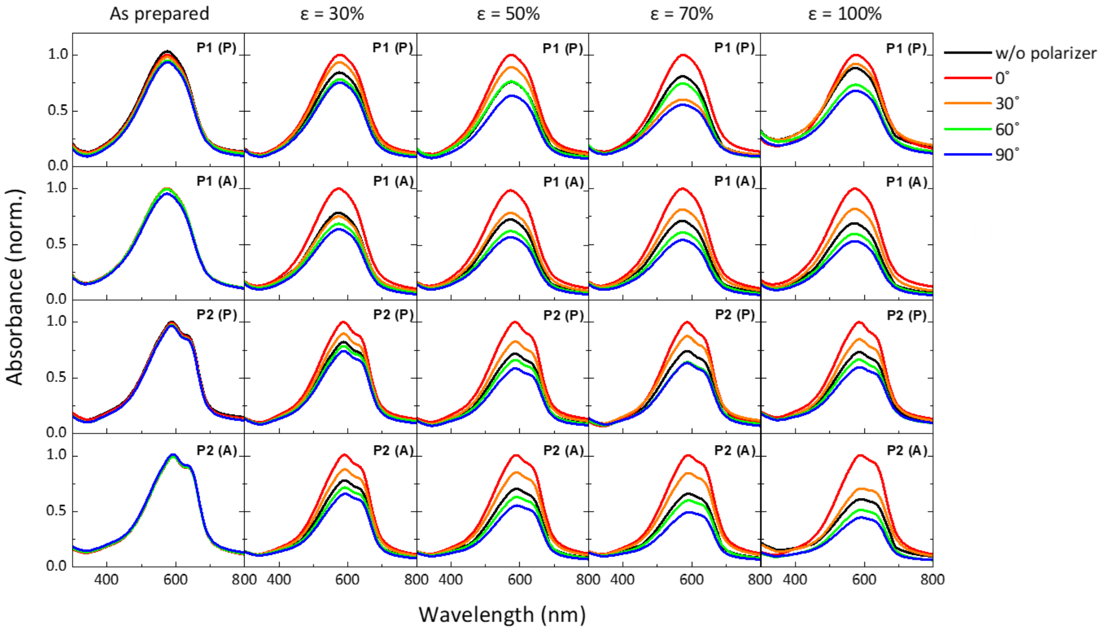

Figure S11. Polarized UV–vis absorption spectra of the pristine (P) and annealed (A) P1 and P2 thin films under various strains (ε = 0%, 30%, 50%, 70%, and 100%) obtained at various polarization angles (θ), where θ refers to the degree between stretching and polarizing directions.

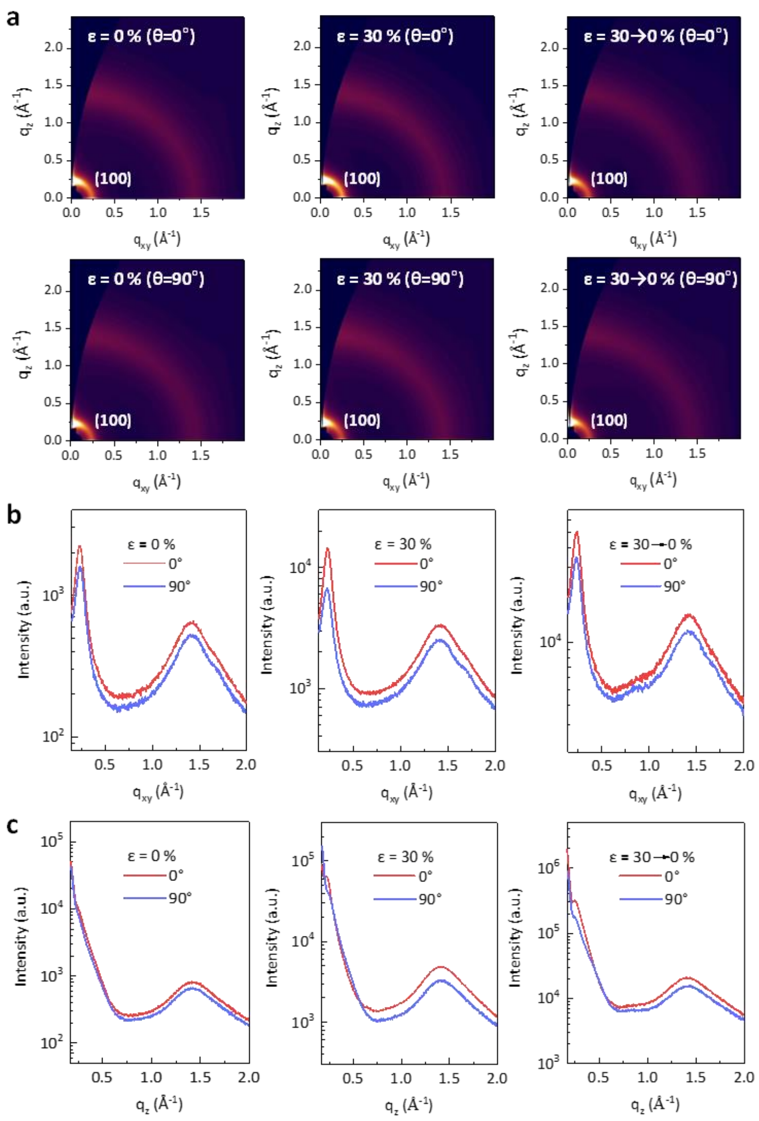

Figure S12. a, GIWAXD reciprocal space maps for the pristine P1 thin films obtained using X-ray beam incidents in various directions under various stretching conditions (ε = 0%, 30%, and 30%→0%). **b–c**, Linecut profiles in (b) IP and (c) OOP directions extracted from the reciprocal space maps shown in Figure S12a.

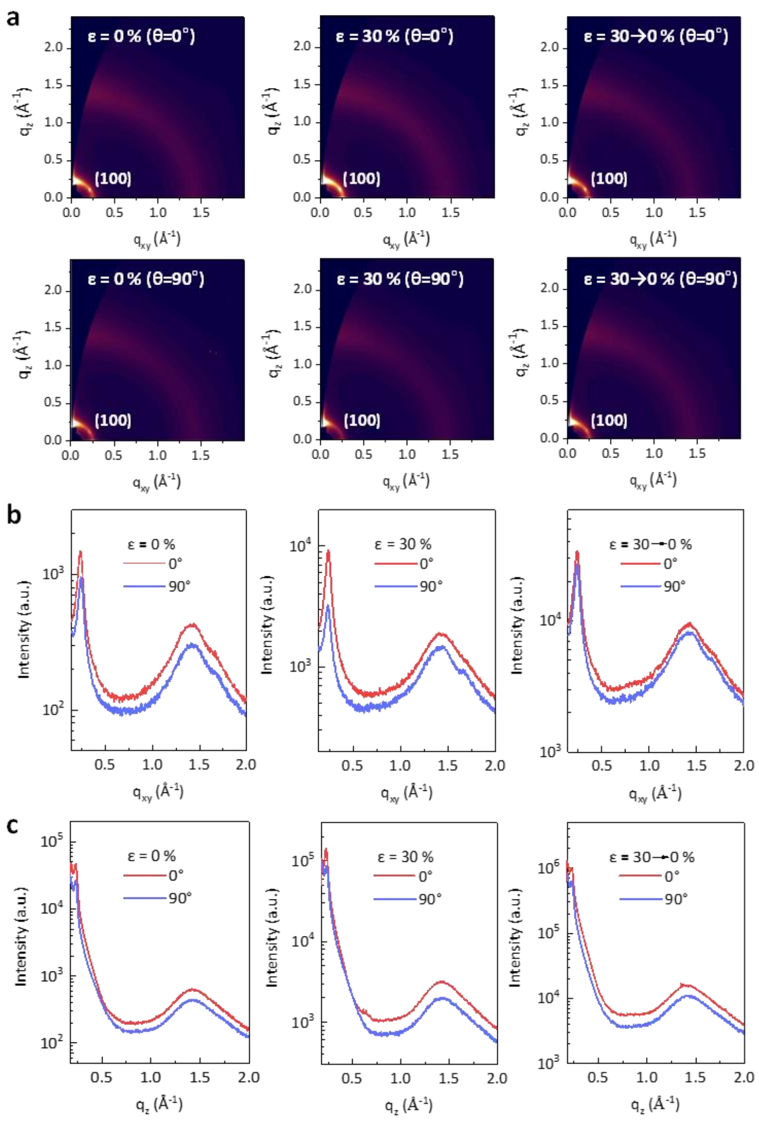

Figure S13. a, GIWAXD reciprocal space maps for the annealed P1 thin films obtained using X-ray beam incidents in various directions under various stretching conditions (ε = 0%, 30%, and 30%→0%). **b–c**, Linecut profiles in (b) IP and (c) OOP directions extracted from the reciprocal space maps shown in Figure S13a.

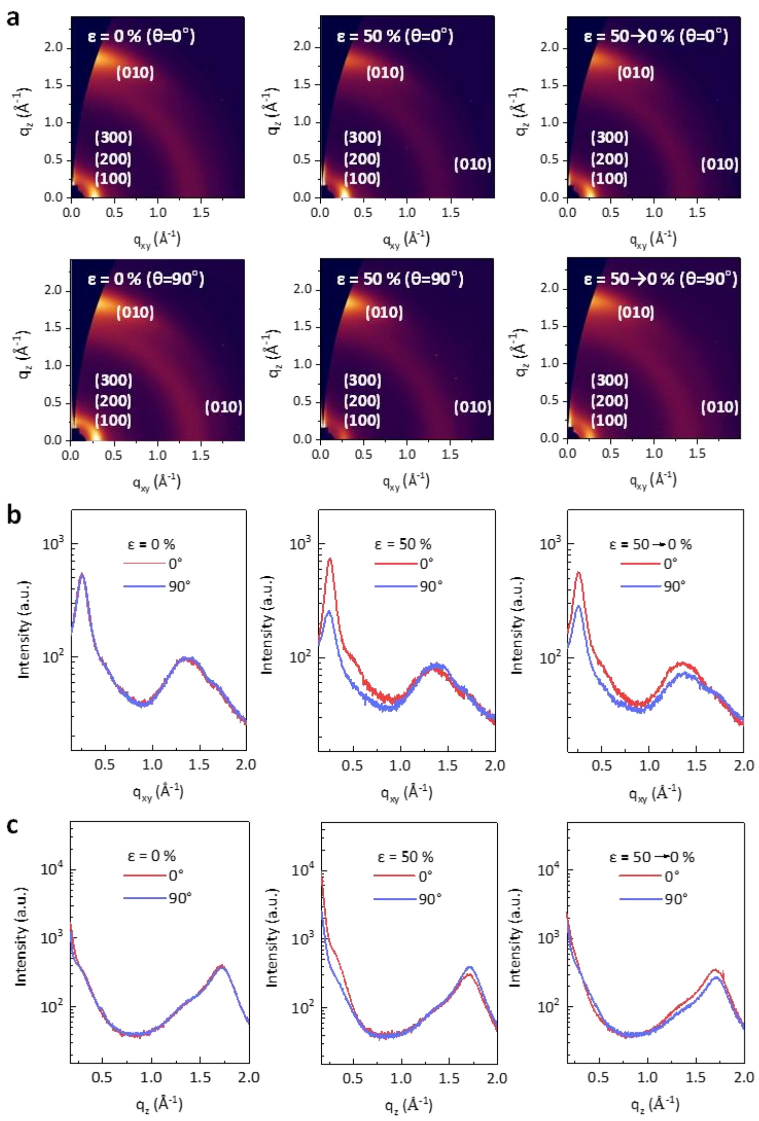

Figure S14. a, GIWAXD reciprocal space maps for the pristine P2 thin films obtained using X-ray beam incidents in various directions under various stretching conditions (ε = 0%, 50%, and 50→0%). **b–c**, Linecut profiles in the (b) IP and (c) OOP directions extracted from the reciprocal space maps shown in Figure S14a.

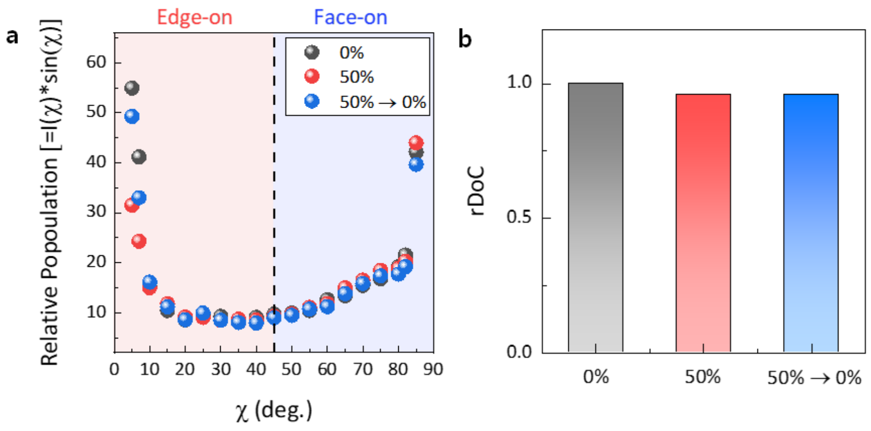

Figure S15. a–b, (a) Relative population of crystallites and (b) rDoC values for the annealed P2 thin films under various stretching conditions (ε = 0%, 50%, and 50%→0%).

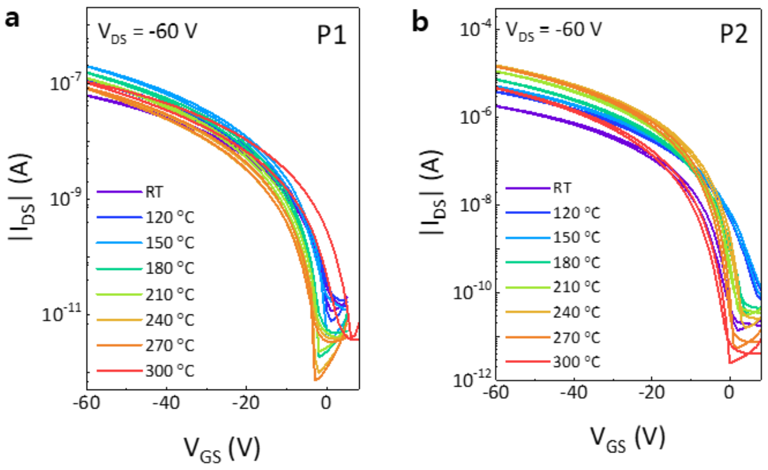

Figure S16. a–b, Transfer characteristics of the OFETs fabricated with the (a) P1 and (b) P2 thin films before (RT) and after thermal annealing at various annealing temperatures, where the P1 and P2 with MWs of 24 and 25 kg mol^{-1}, respectively, were used for this experiment.

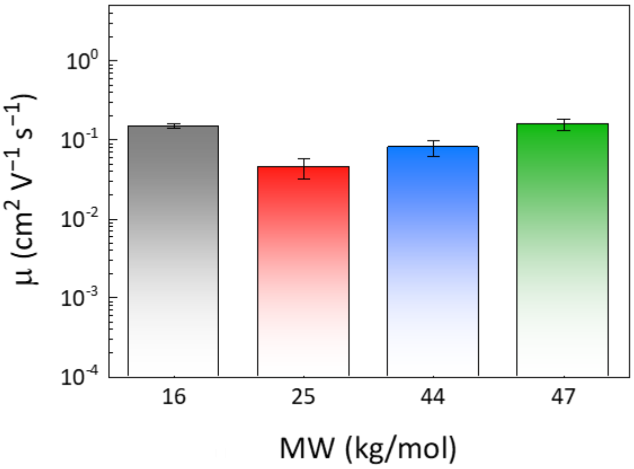

Figure S17. Average charge-carrier mobility (μ) variations of the annealed P2 thin films with various number-average MWs of 16, 25, 44, and 47 kg mol^{-1}.

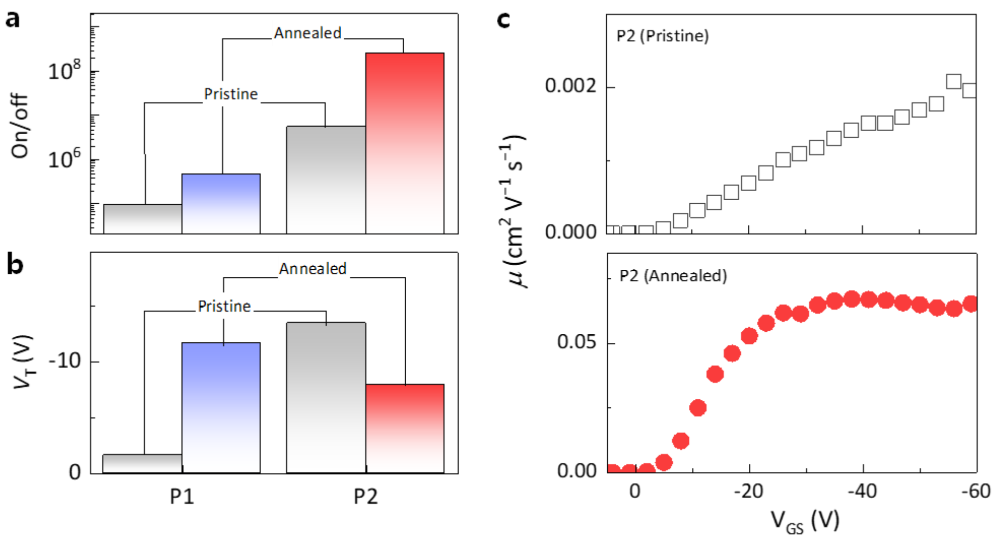

Figure S18. a–b, (a) On/off ratio and (b) threshold voltage (V_T) of the bottom-gate/bottom-contact OFETs fabricated with the P1 and P2 thin films before (pristine) and after (annealed) thermal annealing. **c**, Hole mobility variations of the devices fabricated with the pristine and annealed P2 thin films as a function of applied gate bias (V_{GS}), where the P1 and P2 with MWs of 24 and 25 kg mol^{-1}, respectively, were used for this experiment.

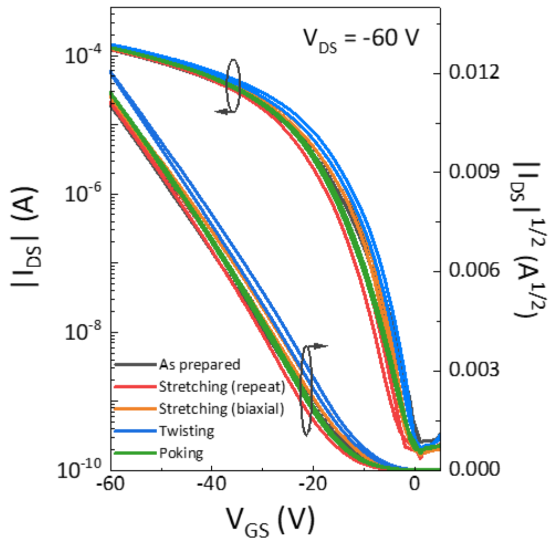

Figure S19. Transfer characteristics of the OFETs fabricated with the annealed P2 thin films under various stretching conditions, including biaxial stretching at ε = 100%, repetitive uniaxial stretching at ε = 50% (×200), twisting, and poking.
